# The use of intuitive AF-based modelling for the understanding of activation and signalling through insulin-like receptors

**DOI:** 10.3389/fendo.2025.1633449

**Published:** 2025-08-22

**Authors:** Andrzej M. Brzozowski, Talha Shafi, Olga V. Moroz, Pierre De Meyts

**Affiliations:** ^1^ York Structural Biology Laboratory, Department of Chemistry, University of York, Heslington, York, United Kingdom; ^2^ de Duve Institute, Faculté de Pharmacie et des Sciences Biomédicales, Université Catholique de Louvain, Brussels, Belgium

**Keywords:** insulin, insulin receptor (IR), IGF, IGF-1R, drosophila, human

## Abstract

The understanding of the conformational events occurring through the Insulin-like Receptors (ILRs) upon binding of their cognate hormones is very advanced, thanks to multidisciplinary efforts and breakthroughs, from molecular/cell biology to structural studies. However, the full length structures of this class of Tyrosine Kinase (TK) receptors are still not available. This is due to fundamental methodological constraints: a need for lipid micelles or nanodiscs required for the stabilisation of the full-length single receptor molecule. They tend to occlude the transmembrane (TM) and intra-cellular parts of the receptors, which, together with their inherent dynamic characters, prohibits - so far - determination of their full, continuous structures. Nevertheless, there is plenty of crystallographic evidences about separate TKs, some also with parts of the Juxtamembrane (JM) region that links the TM helices with the TKs. There are well over 40 known structures of the ectodomains (ECDs) of the ILRs in different complexes with hormones and their analogues, representing a wide spectrum of conformations. However, there is still a remaining question how a particular stage of ECD:hormone binding is translated into activation of the TKs. Here, we attempt to fill this ECD - TM-JM-TK structural gap by employing a simple AlphaFold2-based modelling of these regions, and combining AF2-derived models with the already determined ECD structures. This allows us to propose here a general ILRs activation model where the JM-TK close contacts with the inner leaf of the cell membrane contribute to the activation of the receptors. A possible dual role of the JM region in this process – both TK auto-inhibitory and stabilizing - has been highlighted as well. It also seems that the diverse natures of receptors:membrane lipid interactions require more experimental attention for the full understanding of the signal transduction through Insulin-like Receptors.

## Introduction

**Figure 1:**
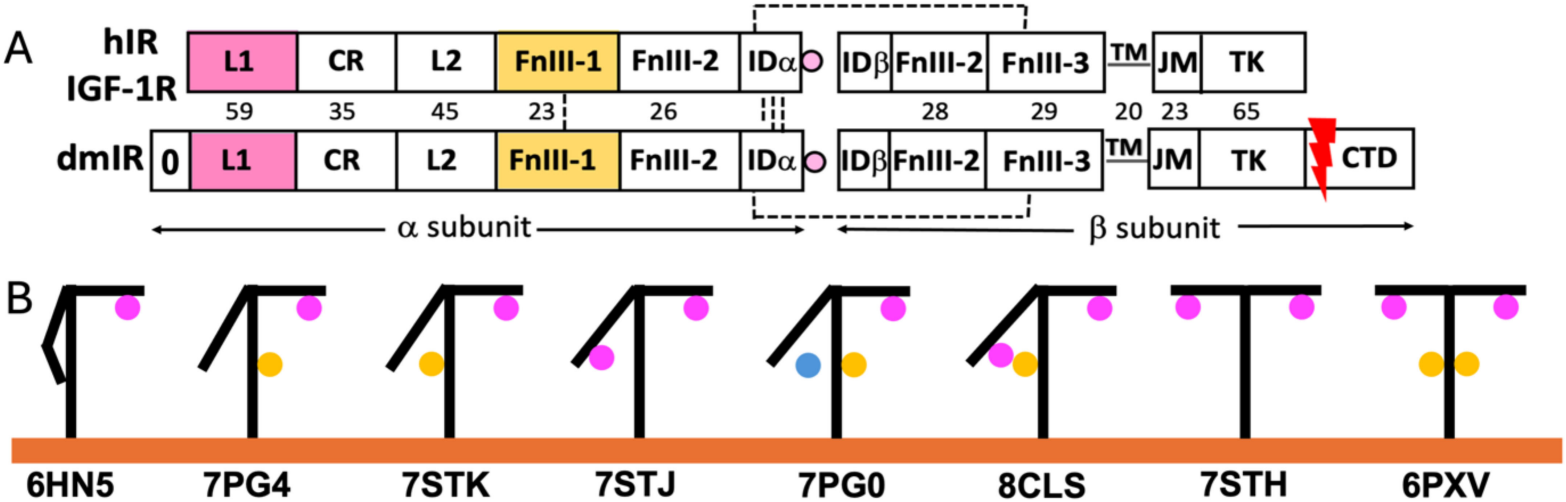
General organisation of the subunits of human Insulin Receptor (hIR), IGF-1R and *Drosophila melanogaster* Insulin Receptor (dmIR) **(A)**, and examples of structural conformers of Insulin-Like Receptors and hormone:receptor stoichiometries **(B, A)** Subunit organisation of the hIR/hIGF-1R (top) and dmIR (bottom) receptors. Hormone Site 1 binding domains (L1 and α−CT terminal helix of the IDα) are highlighted in magenta; main Site 2 binding FnIII-1 domain in yellow. Red arrow indicates processing of the 368 amino acids CTD C-terminal dmIR unique; dmIR N-terminal ‘0’ domain is indicated, but its role is unclear, and it has not been included in any dmIR structural work. Dash lines represent -SS- bridges, and numbers correspond to sequence identities between hIR and dmIR. See main text for the abbreviations. **(B)** Schematic representations of examples of the known structures of receptors ectodomains (ECD) indicating a variety of hormone:receptor stoichiometries; hormones at Site 1 are in magenta, at Site 2 in yellow and between these sites - in blue. PDB IDs of representative structures are given below yellow (membrane) line.

The Insulin Receptor-like (IRL) family of Tyrosine Kinase receptors (TKRs) governs some of the key hormonal signalling with pleiotropic control of metabolism, growth and life span (1–3). All these receptors share very similar (αβ)_2_ subunits, domains and general 3-D structures organisation, comprising of the ectodomain (ECD), transmembrane (TM) and juxta-membrane (JM) regions, leading to tyrosine kinase (TK) which ends with C-terminal tails (**Figure 1A**) (for reviews see (4, 5)). In humans, IRLs include two isoforms of Insulin Receptor (hIR-A, hIR-B (with additional 12 amino acids insert at the ends of the α-subunits)) (6) and Insulin-Like Growth Factor Receptor 1 (IGF-1R). Recent structure of insect *Drosophila melanogaster* IR (dmIR) ECD in complex with DILP5 - one of this insect’s seven insulin-like hormones - revealed its remarkable similar blueprint to human homologous complexes (**Figure 1B**, PDB ID 8CLS) (7). These findings suggest that a general conservation of the IR-like template can be expected in other invertebrates as well, underpinning the molecular and structural bases of a remarkable conservation of the insulin signalling axis in the animal kingdom.

The last ~12 years brought a long-awaited first insight into the structures of human IRs and IGF-1R, firstly crystallographic (8), and, subsequently, by cryo-EM microscopy (9), followed by many of their apo- and holo-forms in complexes with endogenous hormones (insulin, IGF-1, IGF-2), as well as viral insulins, *de-novo* engineered functionally-active (insulin agonists/antagonists) peptides and monoclonal antibodies (reviewed (4, 5). They revealed a wide spectrum of hormone:receptor binding modes, stoichiometries of these complexes (**Figure 1B**), and the adaptability/dynamic character of their ECDs (**Figure 1B**).

The IR/IGF-1R structures point towards the likely key steps of the apo-holo transitions of these receptors, such as: (i) a large (~110 Å) separation of the receptors TKs in the inverted V: ᐱ-like form in hormone-free apo-IRLs that maintains their inactive states, (ii) hormone(s) binding to the receptors’ ECDs triggers their large structural rearrangements, closing the gap between the FnIII-1/2/3 domains of the ‘legs’ of the receptors, resulting in the proximity of the TKs and their activity-releasing *trans* cross-phosphorylation.

**Figure 2:**
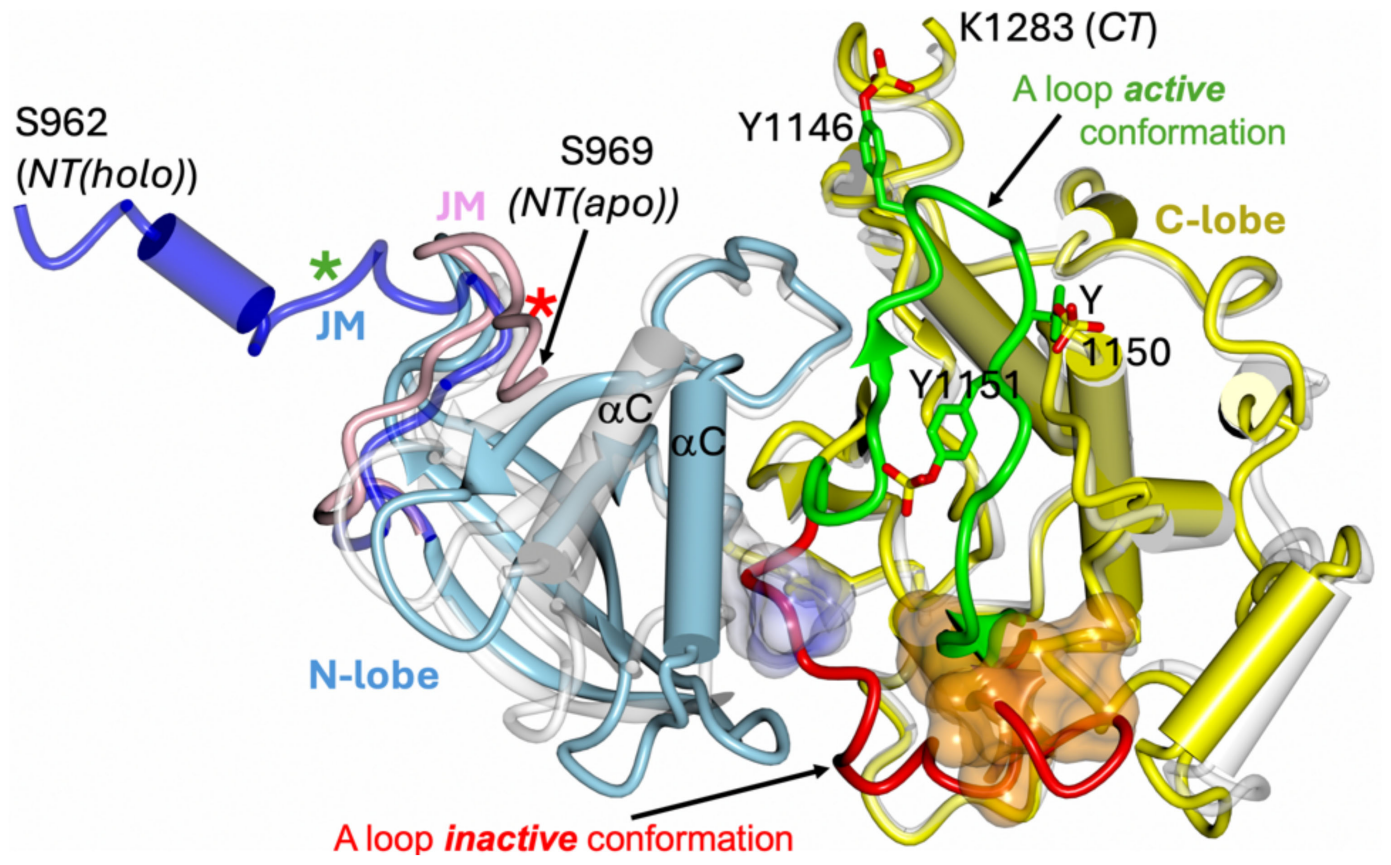
Schematic representation of the inactive and active conformation of hIR tyrosine kinase. The apo TK inactive form (PDB ID 1IRK) (hIRTK-0P) is in white; the holo – active form - of the TK kinase (PDB ID 4XLV) (hIRTK-3P) N-lobe is in blue and C-lobe in yellow. The JM-including segment (962–984) of the holo hIRTK-3P is in dark blue, JM in apo hIRTK-0P (969–984) in pink, and non-hydrolyzable ATP analogue AMPPCP in hIRTK-3P as surface in light blue/white. The six visible residues of 18 amino acid peptide substrate derived from rat IRS-1 determined in the hIRTK-3P:peptide complex (PDB ID 1IR3) are shown as dark yellow surface. The inactive conformation of the activation loop (A loop) in hIRTK-0P is shown in red, with its active conformer in the hIRTK-3P:peptide complex in green. A-loop phosphorylated Tyr1146, 1150 and 1151 (as in 1IR3) are also shown. Stars correspond to position of Tyr972 in the apo (red) and holo (green) TK forms. Please note that TK constructs had mutations that could affect the conformation of the JM/N-terminal parts of these TKs: Cys969Ser, Tyr972Phe (1IRK, 1IR3) and Cys969Ser, Tyr952Phe (4XLV).

**Figure 3:**
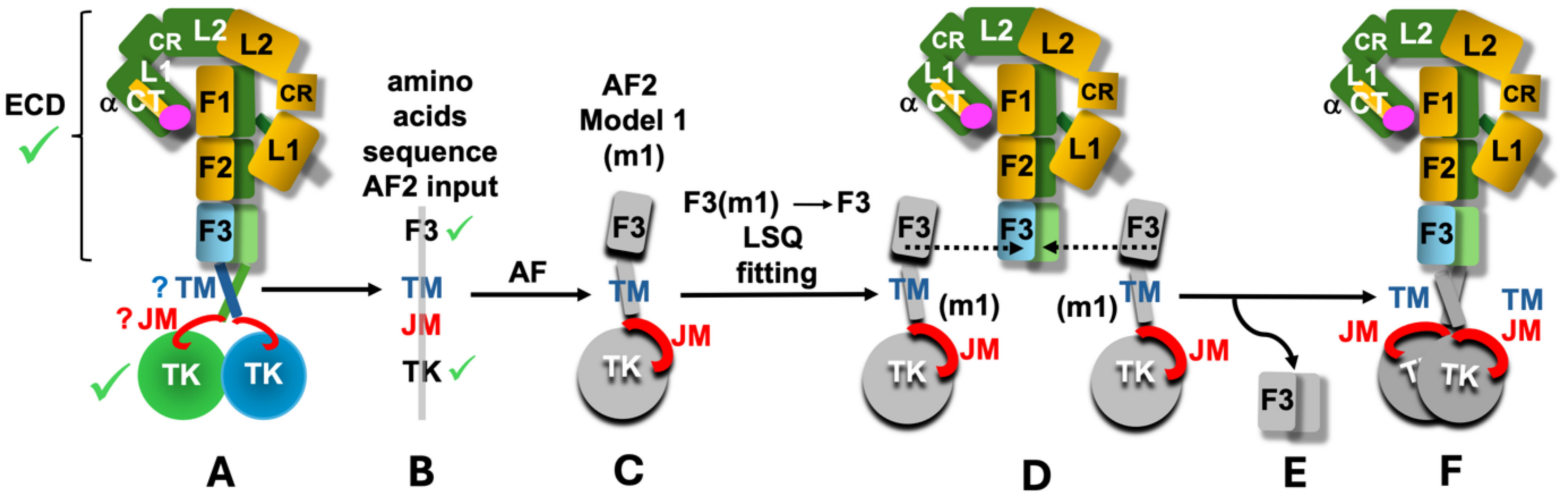
Schematic representation of the main **(A-F)** stages of the performed AF2 modelling exercise. Known 3-D structures of the parts of the ILRs are indicated by green tick (scheme of only one representative example of hIR is used here (e.g., PDB ID: 6HN5/6HN4)). Question marks indicate limited available structural definition of the TM and JM regions; m1 – model 1 (denotes an example of one of the top five models selected here). F1, F2 and F3 are simplified notations of the respective FnIII-1, 2, 3 domains, and the ID segments were omitted for the clarity of the scheme. See main text for details.

**Figure 4:**
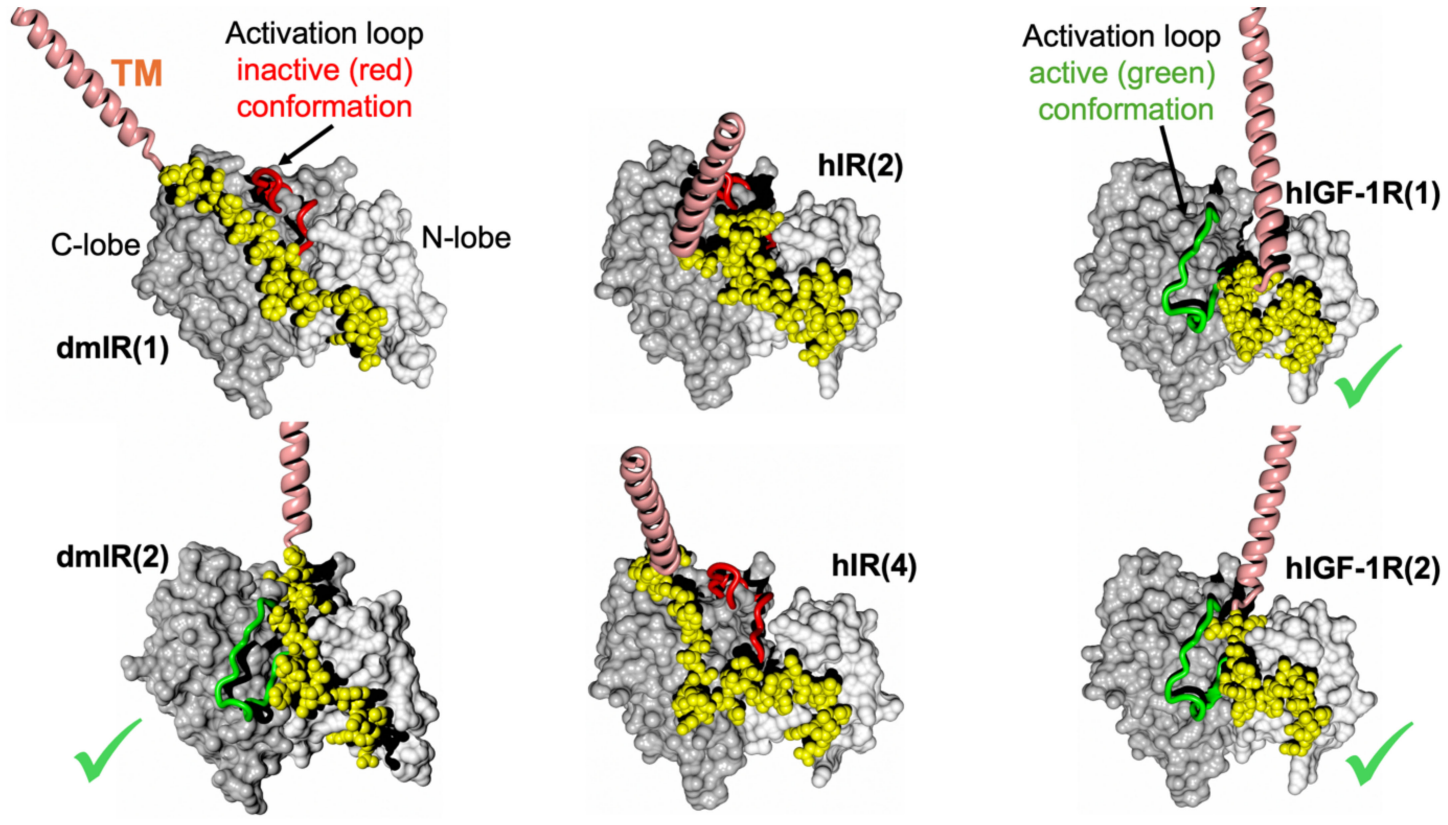
AF models of the TM-JM-TK domains of the dmIR, hIR and hIGF-1R. TKs’ N- and C-lobes are in white and grey spheres, respectively; TM helices are in pink and the JM regions in yellow. The TKs’ activation loop (main chain only) in the inactive conformation is in red, and in its active conformation in green (also indicated by a green tick). In brackets – the number of one of the top five models used here.

**Figure 5:**
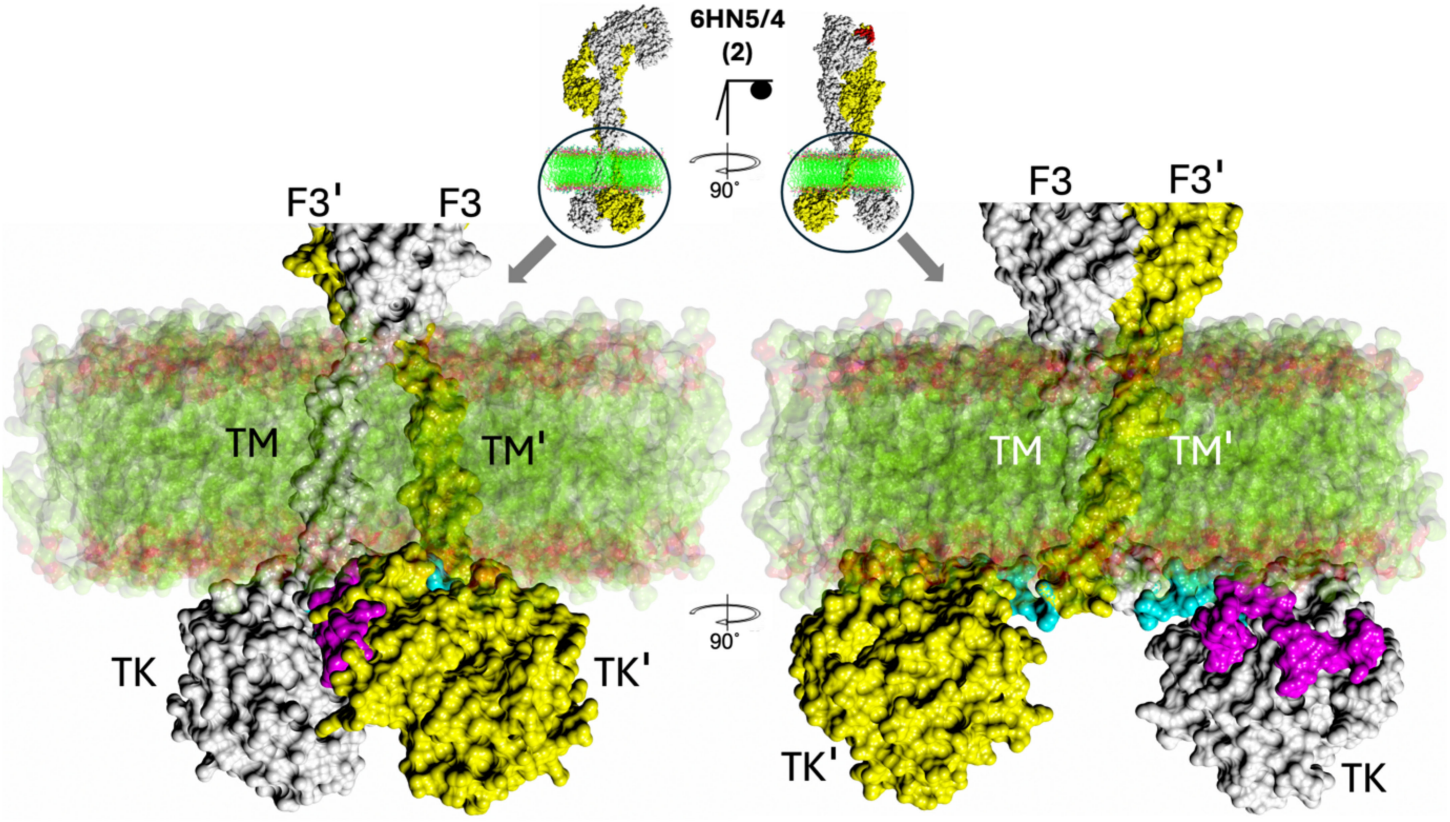
Zoom-in on membrane:FnIII-3-TM-JM-TK surface. The hIR(ECD):one insulin complex (PDB ID 6HN5/6HN4) and AF2 model 2 (centre top) was selected as a typical example of JM-TM:membrane proximity that is typical for all other complexes studied here (see **Figure 6**). F3 – FnIII-3 domain, TM – trans-membrane domain, TK – tyrosine kinase domains. The first, so-called linker, 957–956 residues of the JM region are in blue, the remaining 957–984 JM region is in magenta. Membrane bi-layer model structure (PDB ID 2M20) surface representation is in green (alkyl chains) are in red (polar atoms).

**Figure 6:**
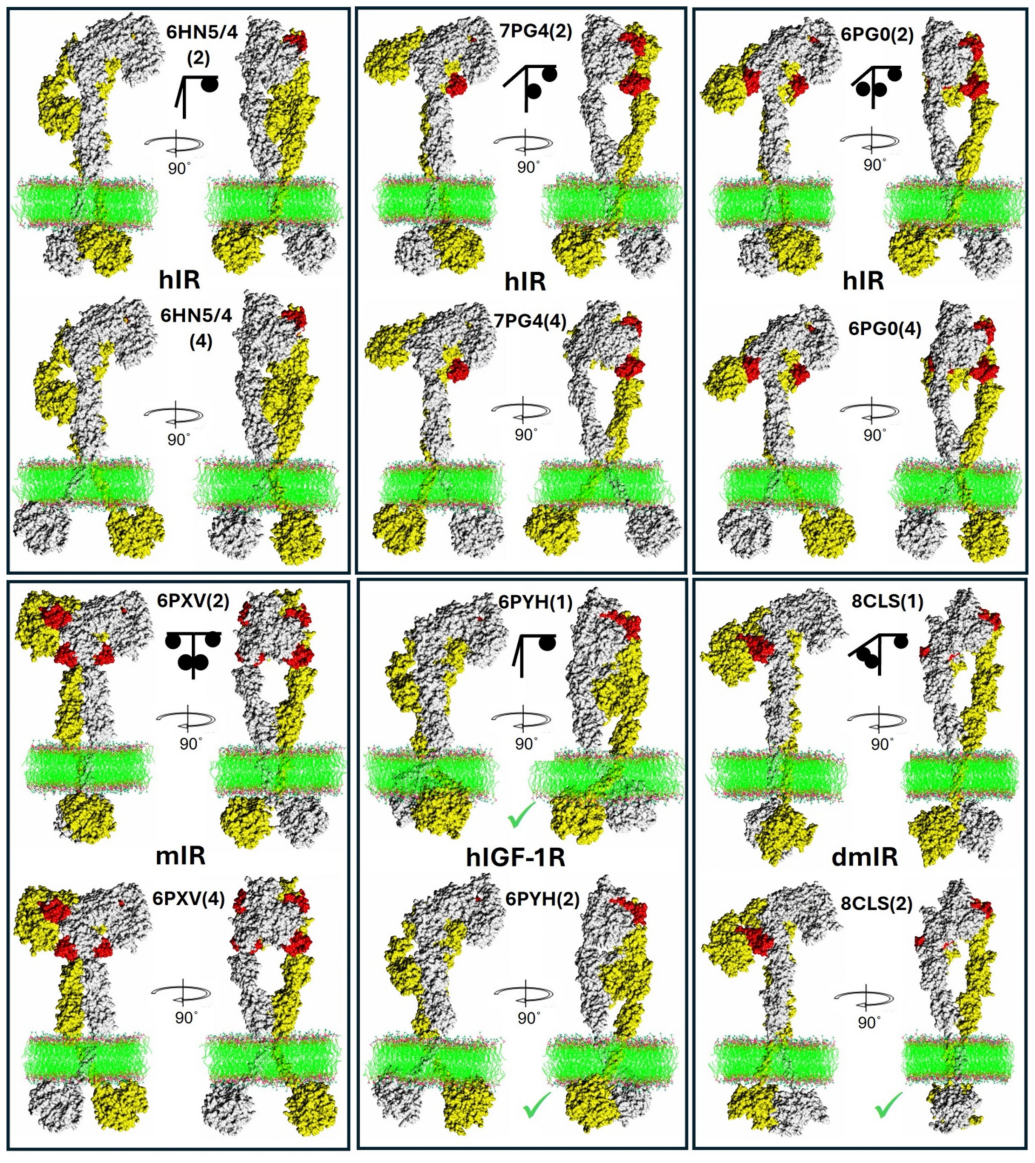
Examples of representative whole Insulin-Like receptors models: experimental ECDs combined with AF2-derived models. Each panel presents the same receptor (in spheres) with its PDB ID and the number (in brackets) of the two best AF2 models used for the two, representative whole ILR assemblies for each particular receptor. The upper *T*-like arm/subunit is in white, the flexible lower arm is in yellow, hormones in red. Lipid bilayer is in ball-stick representation. Green ticks are next to the TKs where the active loop has been predicted in the active conformation (see **Figure 4**). A simple graphical representation of the hormone:receptor stoichiometry is given as a black stick/ball scheme.

**Figure 7:**
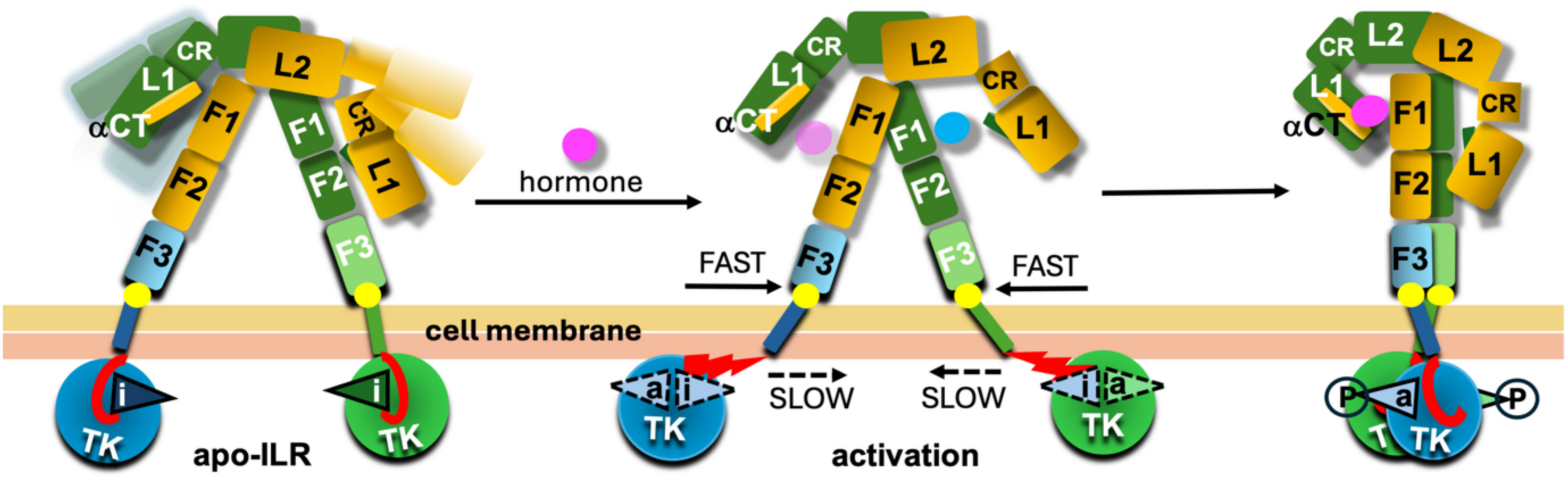
Schematic representation of the drag-friction activation model. The likely dynamic character of the apo-ILR is depicted with a range of conformers of the L1-CR-L2 arms of the receptors in lighter colours. The activation loops are given as triangles with ‘i’ and ‘a’ indicating their inactive or active conformations, respectively. The dynamic character of these loops upon activation (central panel) is indicated by dashed contours; circled P symbolises phosphorylated states of these loops in their active conformations. The JMs regions are in red, and their unzipping from the TK’s groves between N-/C-lobes upon activation of the TKs are given as staggered red triangles in the middle panel/conformer. FnIII-3 (F3) C-terminal Proline ‘pivot’ on the membrane interface is depicted as a bright yellow circle. The hormone in magenta and blue reflect the postulated migration of the hormone from site 2 (blue) to final site 1 (magenta). Two types of yellow colours reflect the chemical and structural differences between the inner and outer leaves of cell membrane.

In one-insulin:hIR (and one-IGF-1:IGF-1R) complexes the hormones are bound to the upper arm of the hIR in *T*-like conformation (so-called high affinity site 1), while the other arm remains close to the stem of the receptor formed by FnIII-2/FnIII-3 domains (**Figure 1B** – e.g. PDB ID 6HN5/4) (e.g (10–14). Such hormone binding mode is sufficient to bring together (~30-10 Å) the membrane-proximal FnIII-3/3’ domains, hence, possibly, translating these close contacts onto an ‘active’ engagements of the TMs, JM and TKs part of the IRs ((**‘**) denotes corresponding sites in the other subunit)). On the opposite range of insulin:hIR complexes, four insulins saturate the almost symmetrical T-like conformer of the receptor by binding to both sites 1/1**’,** and to so called low affinity sites 2/2**’** that are located on the FnIII-1 (and parts of the FnIII-2 in dmIR) domains (**Figure 1B** – 6PXV) (15) (16). Structures of many other conformers of the complexes have been determined with two, three and four insulins in one receptor; they usually appear in often subtle variations of asymmetrical *T* and *T* -like conformations, with different occupancies of 1/1**’** and 2/2**’** sites, and, in some cases, with insulin ‘bridging’ the lower arm and the stem of the IR, not being fully engaged with either site 1 or site 2’ (**Figure 1B** – 7PG0) (17). Overall, site-1/site-2 binding modes correspond to a well-established two different receptor-binding epitopes on hormones’ surface (18). Interestingly, hIGF-1:IGF-1R complex has only been obtained with one IGF-1 bound, with the α−CT helix threading through the IGF-1 C-domain-forming loop that joins in this hormone equivalents of insulin’s B- and A-chains (11). The first invertebrate dmIR-ECD structure in complex with three insect DILP5 insulins also falls – in general – into the landscape of hIR/IGF-1R conformations, despite that two DILP5 hormones are sitting on the lower arm in close contacts neighbouring site-1 and site-2**’**, while the third DILP5 occupies the classical site-1**’** on the *T*-like upper arm of the receptor (**Figure 1B** – PDB ID 8CLS) (7).

The intracellular TK components of the IRLs have also been studied extensively, resulting in many 3-D structures of their both inactive and active conformations (19–25), allowing outlining a general catalytic and activation mechanisms of these kinases (26–29). The TKs of the ILR receptors are very homologues, following also a very similar 3-D structural blueprint, with a typical protein-kinase fold, which is also greatly maintained in monomeric classes of the TK receptors (e.g., EGFR, Ron, c-Kit etc.) which dimerise upon hormone binding (30) (**Figure 2**). Both human hIR and hIGF-1R TKs have typical two domains - N- and C-terminal lobes – organisation (19, 22), with the C-lobe containing most of the active site residues and so-called activation loop. The activation of the ILR’s kinase involves *trans*-phosphorylation of three tyrosine side chains of the activation loop, and its flipping over, revealing the active, ATP- and substrate binding sites which are occluded by this loop in the kinase inactive state (28) (**Figure 2**). TK activation relies also on a significant closure of the N- and C-lobes, that results – among other structural changes – in the shift of so-called αC helix that places then catalytically important residues close to the ATP binding site. (**Figure 2**). Subsequently, three tyrosines in the C-terminal tails of the TKs, and two in the JM region can be phosphorylated for the recruitment of the key downstream signalling effectors (e.g., Insulin Receptor Substrate-1/2, Shc, SH2B1, SH2B2), or for the internalisation of the receptor (31–33). It is also envisaged – mostly *via* biochemical evidences - that the kinase-proximal part of the JM region (still very elusive in structural studies) plays a TK auto-inhibitory role, maintaining ILR basal, quiescent state (34–37); this was also confirmed in some apo-monomeric type receptors’ TKs, such as in the EGFR (38–50). Structural studies of this mechanism in the hIR showed that JM Tyr972 – conserved in all ILRs in the animal kingdom – interacts in the TK apo state with also conserved residues of kinase’s N-lobe, stabilising the αC helix in a catalytically non-productive conformation (36) (**Figure 2**).

Insect *Drosophila* dmIR has a unique additional ~368 amino acids extension of each TK (with some homology to the IRS-1), which can be processed (cleaved off) in certain tissues (51)(**Figure 1A**); its role is still not fully known.

The exact mechanism of activation of the whole hIR/IGF-1R, and dmIR upon hormone binding has not been clearly visualised, but structural and functional data point towards the *trans*-phosphorylation of their TKs being brought together upon transition from apo-to-holo states of the receptors. It seems that the TKs *trans*-phosphorylation of ILRs follows, to some extent, a symmetrical pattern, which is in contrast to some monomeric TK-receptors, e.g., EGFR, in which ligand-induced dimer only one kinase is active upon *cis*-phosphorylation, being allosterically stabilised by TK from the associated receptor subunit (aka CDK: CD-like functioning) (52).

Despite recent astonishing progress and breakthroughs in the structural/functional characterisation of ILRs, the insight into the actual structural path of signal transduction through these receptors is still rather binary, with separate focuses on the roles of ECDs and TKs in this process. This extra-/intra-cellular division of our understanding of the IRL receptors results mainly from current methodological constraints. The studies of ECD-to-TK signal transduction are hampered by the occlusion of the transmembrane, JM and TK regions by lipids necessary for the extraction and stabilisation of the whole ILRs, as well as due to an inherent dynamic character of these downstream components of the receptors (16, 53).

Such methodological challenges lead to alternative views about the nature of the ‘native’, transmembrane signal-inducing ILR ECD conformer (e.g (17, 54). However, it can be envisaged, that considering the low pM concentrations of circulating insulin (going potentially towards nM range after meals), one-insulin:IR asymmetric complex should suffice to activate the whole receptor. It may emerge in this state *via* a transient low-affinity site 2 complex which initiate destabilisation the apo - ᐱ-like (or dynamically similar) – IR conformer (5). However, this single-hormone bound receptor model supports well IGF-1:IGF-1R as the main signalling conformer, but it does not fully explain the overall sub-nM (~0.02 nM) high hIR affinity of insulin, not covering quite the interactions of low affinity (~0.2 nM – 2μM (hIR construct dependent)) sites 2 on insulin surface and the hIR (17, 55) Structures of two-insulins:IR complexes can correspond to some transient states towards the final single-molecule assembly; although three/four-insulins:IR complexes are rather unlikely prevailing native forms of the hIR due to a limited physiological presence of the hormone, they cannot be excluded.

Therefore, prompted by the still existing structural gap between ILRs’ ECDs and their TKs we attempted to probe whether the use of AlphaFold2 (AF2) (56) can provide more structural and functional insights into bridging the knowledge about the ECDs and TKs of these receptors, by shedding some light on possible 3-D arrangements of the TM, JM and TKs modules, in the context of different, known structures of Insulin:IR (and IGF-1:IGF-R) complexes. NMR structure of IR TM helices in lipid micelle is available (57), and the longest known structure of the JM segment was described in the IGF-1R apo-TK mutant (referred here as to IGFRTK-0P, PDB-ID: 1P4O) which starts at Pro948 (Pro958 in IR-A isoform of which numbering is used through this text) (58), while the whole hIGF-1R JM spans ~Asn934-Ile969 (app. ~Pro945-Ile984 in hIR-A, including first 12 linker regions in both receptors). However, the JM chain is stabilised in IGFRTK-0P by the crystallographic dimer interface between the kinase molecules. Similarly, the longest structurally-visible JM in the hIR – from Ser962 onwards - has been observed in the crystal structure of the holo-dimer of triple-phosphorylated TK (IRTK-3P, PDB ID: 4XLV) (24). Hence, the known conformations of some parts of the JMs of IL receptor can be biased by crystal packing-related inter-molecular contacts.

Therefore we undertook a simple exploratory modelling exercise where we employed the AlphaFold2 (56) to predict the structures of FnIII-3-TM-JM-TK parts of the hIR, hIGF-1R and dmIR, superposing subsequently the AF2-derived FnIII-3 domains of the FnIII-3-TM-JM-TK segment on their counterparts in the experimentally determined representative ECD conformers of these receptors (**Figure 3** – see details in the following chapter). We expected that the relevance of these FnIII-3-TM-JM-TK AF2-derived models can be enhanced by excellent, known 3-D templates for the flanking FnIII-3 and TK domains, for which AF2-generated folds obtained here should be very accurate, and ‘native’-like. Although AF2 predictions discussed here lacks 100% reproducibility, i.e., their repetitions always result in an overall slightly different relative spatial distribution of FnIII-3-TM-JM-TK domains, the key motifs of these models (e.g., JM engagement with the TK discussed below) are still present, and are – overall – consistently repetitive.

The key questions we tried to address here were as follows: (a) whether the AF2 can help to correlate the orientations of the FnIII-3/3’ domains observed in the structures of different IR and IGF-1R complexes with the orientations of the downstream TM-JM-TK parts of the receptors, (b) whether the AF2-driven FnIII-3-TM-JM-TK modelling, and the fusion of these models with the experimental receptors’ ECDs structures, is capable of revealing any new - potentially *in vivo* significant - types of interactions in the TM-JM-TK regions, especially of the ‘elusive’ JM domains, and (c) whether the full length IR and IGF-1R conformers observed in combined true-ECD-structures+AF2-models can shed any light on the receptor activation process, i.e., signal transduction through these receptors, which still awaits unambiguous, experimental structural elucidation.

## AF2 modelling exercise

The models of the FnIII-3-TM-JM-TK segments of hIR, hIGF-1R and dmIR were obtained by the AF2 programme in the CCP4 Cloud (59) for the following IRL amino acid sequences: hIR - Val801-Ser1343, hIGF-1R – Val791-Cys1337, dmIR – Tyr1191-Gln1828. The TK-following dmIR-unique C-terminal Domain (CD domain) (**Figure 1A**) was not included here as (a) it was clear after initial trials that the AF2 was not able to predict its relevant structure (as expected: no known-template for CD sequence in the databases), and (b) its occurrence is limited exclusively to dmIR. The flow of the whole modelling process in outlined schematically in **Figure 3**. Firstly, the representative ECD structures of the ILR-family, with different hormone:receptor stoichiometries and variety of conformers (from one arm up/one fully down to almost symmetrical T-state ones) (**Figure 1B**) were selected (**Figure 3**– stage A. Next, the abovementioned sequences of the receptors’ FnIII-3-TM-JM-TK regions were submitted for the prediction of their structures to the AF2 programme (**Figure 3** – stage B). AF2 produced several models for each FnIII-3-TM-JM-TK sequence, and top five for each receptor (m1-m5) were used for further analysis (**Figure 3**– stage C). Subsequently, the FnIII-3 domains of the AF2 models m1-m5 were superposed on their corresponding FnIII-3 and FnIII-3**’** equivalents in each representative receptor ECD in programme COOT LSQ option, on their Cα atoms (60) (**Figure 3**– stage D). The Cα target atom ranges for these superpositions was Asp813-Val905 for the hIR, except for 6HN5/6HN4 structure (10) with a different numbering where models Ala811-Thr906 were superposed on 6HN4/5 Ala792-Thr887, while for the dmIR this FnIII-3 Cα target atom range span Asp1209-Val1302 for both models and the dmIR. For the mIGF-1R:hIGF-1 structure (PDB ID 6PYH) (14) the Cα range for the FnIII-3 domains was Gly800-Phe893 that were superposed with models Gly801-Phe892; this was due to differences between the structure and model sequences here (6PYH is a highly homologous mice IGF-1R (mIGF-1R) (96% seq. identity with the hIR) complexed with hIGF-1).

Subsequently, after these superpositions, the redundant/duplicated now pairs of models’ FnIII-3/FnIII-3’ domains were deleted in COOT (**Figure 3**– stage D), yielding a ILR’s full-length ‘hybrid’ model, comprising of an experimentally-known ECD structure of a particular receptor that was followed by the AF2-modelled TM-JM-TK (**Figure 3**– stage E). Finally, these full-length IRLs models were manually placed (translate/rotate option in COOT) in a phosphocholine lipids bilayer: one of the typical models of human cell membrane - derived from the NMR structure of the hEGFR TM helices determined in these lipids (PDB ID 2M20). Lipids DMPC-to-DHPC ratio of in the 2M20 was 1:0.25, where DMPC is 1,2-ditetradecanoyl-*sn*-glycero-3-phosphocholine, and DHPC - 1,2-dihexanoyl-sn-glycero-3-phosphocholine (61). Finally, some lipid molecules were deleted in COOT to accommodate TM segments. Analysis of the models have been performed in COOT, and the best models, i.e., without steric clashes, were finally analysed, and presented here in figures made by the CCP4MG programme (62).

It should be noted that the conformation/proximity of the Fn-III-3 domains, and, in general, overall conformations of the ILRs’ stems, hence the structure of the full receptors’ models presented here, could be affected by different tagging of the C-termini used for the expression and purification of a particular receptor construct. Here, the C-termini of the FnIII-3 domains were as follows: PDB ID 6HN5/6HN4 – hIRA ECD (1–916) ended with 33-residue GCN4 zipper sequence (RMKQLEDKVEELLSKNYHLENEVARLKKLVGER), and with Y144H, I421T, and Q465K mutation; Fv83–7 monoclonal antibodies were used for complex stabilization (10); 7PG4 – was a full-length hIRA with human protein C-tag (17); 7PG0 – the same as for 7PG4; 6PXV - full-length hIRA with endocytosis-preventing mutations Y960F, S962A, D1120N, R1333A, I1334A, L1335A, L1337A, untagged (Tsi3 and His8 tags were removed during purification) (16); 6PYH – *Mus musculus* IGF-1R (mIGF-1R), full-length with C-terminal truncation (1–1262), and Y951A, D1107N kinase internalization-preventing mutations (Tsi3 tags removed upon purification) (14); 8CLS – dmIR ECD (264-1309), C-terminal StrepII-tag (7). Nevertheless, despite the C-terminal differences in these constructs, their consistently recurring structural convergences, i.e., similar proximities of their FnIII-3 domains in their full-length, or ECD, liganded states, allow to assume a considerable physiological meaning of these conformations.

## Results and discussion

Overall, the LSQs-derived rms for the corresponding structures with known-models of FnIII-3 and TK, were very low - in the range of ~0.8-0.9A - as the AF2 predictions of these domains were very accurate due to their many known 3-D templates/structures. The dmIR TK was also predicted with an excellent accuracy due to its very high homology with hIR and hIGF-1R TKs (~60% identity), and its similar AF-based model has already been analysed (25). The trans-membrane sequence peculiarity of the TM helices, together with some known NMR structures (57, 61), resulted in their typical helical models, as expected.

The AF2 predicted local difference test (pLDDT) scores (0-100, score >80 shows high confidence of the prediction) were low, usually below 50, for the TM, JM and TKs’ active loops regions, remaining well >80 for the remaining parts of the proteins. However, a fully truthful conformations of these ILRs’ regions was not really expected, but rather their possible 3-D arrangements in the context of well known, hence reliably predicted, flanking FnIII-3 and TK domains.

The most anticipated predictions here were the putative folds of the JM regions, especially in the context of their neighbouring TM and TK domains, as their available 3-D definitions are very limited. As mentioned above, the JM hIR region is structurally best defined in hIRTK-3P crystal structure (PDB ID 4XLV) (24) – from Ser962 onwards (IR-A notation), but it points away from the TK’s core, being trapped in crystal-symmetry JM-JM contacts from the other TK, and is also wedged there between the β-sheet and alpha-helix (αC) surface of the second TK molecule (residues 944–961 are disordered/invisible there). This JM conformation is also stabilised there by the interaction of its N-terminal part with the third TK crystal-symmetry related molecule. The JM region from Val966 is also visible in the crystal structure of the inactive hIR TK (PDB ID 1P14) (36), where the Val966-Glu976 JM chain – in contrast to hIR TK-3P (PDB ID 4XLV) structure (24) - runs close to the core of the TK. This work also provides an evidence for the auto-inhibitory role of Tyr972 which interacts there with several conserved residues of the TK’s N-terminal lobe, stabilising a catalytically nonproductive position of the αC helix. The best 3-D evidence for the conformation of the hIGF-1R Pro948-Tyr957 JM segment is in the apo-form of this TK, but it is an important part of the TK-dimer inter-molecular interface (58). It’s worth noting that the JM segment is shorter in the IGF-1R TK, missing the equivalent of Val966-Cys969 peptide present in the IR-A were unpaired Cys969 is frequently mutated in structural studies to avoid protein non-specific aggregating.

Here, the top five FnIII-3-TM-JM-TK models for each AF folding exercise were analysed for packing against the lipid cell membrane model, and potential clashes with kinase counterparts from another subunit of the receptor. Models with the TKs domains too much embedded into the membrane due to a very ‘parallel’ run of their TMs along the lipid bilayer, or where TKs were significantly overlapping, were discarded.

Interestingly, the activation loop was predicted in both active and inactive conformation for the dmIR TK, while it was always in the active conformation in hIGF-1R TK models, and an inactive one in the hIR models.

Also, our attempts to predict the FnIII-3-TM-JM-TK dimers in the next generation AF3 programme (63) were unsuccessful, not yielding any sensible structures. Even more surprisingly, similar AF3 attempts to generate TK dimers (i.e., TKs without any other domains) also failed, despite many excellent structural templates.

### The AF2 predicted folds of the JM region

The first, striking correlation of the conformations of the JM regions was their trend to run - practically immediately after the TM helix - in the groove between N- and C-lobes of the TKs, and close to the surface of the kinase (**Figure 4**). The JMs chains were either very extended (dmIR, hIR) or more compact (hIGF-1R), with their C-terminal/distal parts wrapped around the N-lobe of the TK – as observed in some crystal structures. Interestingly, the in-groove JM conformation was observed for both the active and inactive position of the activation loop. This suggests that the already postulated autoinhibitory role of the JM (e.g (36, 37) could also be expanded to its opposite functionality, involving stabilisation of the activation loop in its flipped, active conformation. Hence, the JM region may possess double, Janus-like properties: it may function as an auto-TK-inhibitory element (already well documented (see above)), or, following receptor activation, it may contribute to the maintenance of TKs activity, thereby supporting prolonged downstream signalling.

We refrain here from discussing structural details, e.g., hydrogen bonds and other JM-TK interactions, as this may be an over-interpretation of the AF2 models. However, JM important Tyr953/965 (IRA/IRB notation), Tyr960/972 and Tyr972/984 are generally involved in contacts similar to those described elsewhere (36, 37). Interestingly, the NMR and functional studies of the Tyr953-Tyr960 octapeptide from the hIR JM region suggested its β-hairpin-like conformation and IR-internalisation signalling role (64); indeed, such β-hairpin was observed in some hIR AF2 JM models (especially model 1), providing some additional retro-experimental validation of the models discussed here and their likelihood.

### The relation of the experimentally-observed FnIII-3/3’ conformations to the 3-D organisation of the downstream TM-JM-TK modules

The analysis of this aspect of our modelling was performed for selected, representative variety of full-length AF-derived hybrid hIR/mIGF-1R models, embedded manually in the widely used model of the lipid bilayer (61) (PDB ID 2M20). Several observations can be outlined here. (i) Placements of the IRLs’ full-length hybrid models into the model of human cell membrane indicate a possibility of very close contacts of the JM-TK with the inner leaf of the lipid bilayer (**Figure 5**). (ii) The respective conformations of the FnIII-3/3’ (i.e., the nature of their relative orientation and proximity) **-** at the membrane-proximal regions of the experimentally determined 3-D structures of the ILRs’ ECDs - do not show here any clear correlation with the ligand:receptor stoichiometry, and the wide dynamic ranges of the lower/upper arms of the receptors (**Figure 6**). (iii) Predicted TM helices are not clustering in one, closely intertwined-like pairs, but show many orientations, some relatively straight through the membrane – some being more parallel to the lipid bilayer. It must be stressed, however, that the variety of TM pairings obtained here should be treated with caution, as they can result from the lack of an external biochemical constraints (i.e., lipid bilayer) during the AF2 modelling process. One of the other reasons of their wide conformational heterogeneity – and probably more physiologically relevant - can be the presence of conserved proline residues at the C-terminal end of the FnIII-3/3’ domains. It is present at these ends of the stems of the receptors in practically all ILRs, making a very specific and narrow point of ECD:membrane contact that is both conformationally robust due its strong ring structure, but simultaneously capable of isomerisation and occupying rather narrow, but very different regions of the Ramachandran plot.

### The IR/IGF-1R drag and friction activation model

The AF modelling-based observations highlighted here tempted us to propose a very general IR/IGF-1R activation model which combines experimental structural evidences with the AF2 models (**Figure 7**). This ILRs activation model assumes following steps of this process. (i) Ligand binding to the apo-receptors is causing conformational changes in their ECDs leading to bringing together their FnIII-1-3/FnIII-1**’**-3**’** subunits. (ii) The FnIII-3/3**’** terminal proline residues are pivots of this movement; while facilitating a conformational stability (i.e., low protein:lipid friction point) for the FnIII-3/3**’**:outer-membrane leaf contact site, they are also able to isomerise/flip into other typical proline stereochemical region if necessary. These proline’s properties (frequently used in proteins’ structural mechano-sensing switches (e.g (65, 66)) can enable quite fast, but controlled (e.g., by lowering random flexibility of the site) getting-together movements of these parts of the receptors. (iii) In contrast, close contacts of the JM/TK domains with the inner leaf of the cell membrane (**Figures 5**, **6**) can result in a substantial molecular protein:membrane friction on this intra-cellular receptor:membrane interface, which, subsequently, is slowing down the dragging of the TKs and their gap-closing movement. (iv) This molecular friction, and the difference in dragging forces between outer-intra-cellular parts of the receptors at their membrane interface, can lead to the unzipping of the JM protein chain from its in-TK-grove, their activation-loop blocking conformation, allowing subsequently flipping of these loops that may be synchronised with their *trans*-phosphorylation in final TK-dimeric-like receptor activation stage (**Figure 7**). The discrepancies between outer-inner membrane leafs dragging forces can be amplified further by their chemical heterogeneity, with the inner leaf being usually a denser one (67–73). The postulated correlation between Type 2 Diabetes (T2D) and some ‘degeneration’ of cell membrane (e.g., its increasing denser/rigid character) (74–76) can also be – to some extent – highlighted by this model. The disturbance (e.g., decrease in T2D) of the JM/TK:membrane ‘friction potential’ can contribute to a lower efficiency of activation of the TKs. Interestingly, conformations of the JM chains in the DFA model can be relatively similar (i.e., in-between TKs’ lobes) in both apo- and holo-forms of the receptors, giving the JM polypeptide chains a Janus-like function in receptor activation.

It should be noted that the proposed DFA activation model – for the sake of its simplicity and a very general character – is based on one, identical FnIII-3-TM-JM-TK structural AF2 model which is then being fitted onto FnIII-3 and FnIII-F3’ receptor’s ECD domains. Hence both β-subunits’ TM-JM-TK parts of a full-length receptor model - downstream from its FnIII-3/3’ domains - are structurally identical. Certainly, different combinations of models, e.g., model 1 and model 2, could be fitted onto FnIII-3/3’ domains respectively; this would lead though to plethora of permutations of not very informative IRLs models, generating unnecessary at this stage, and too speculative, complexity of the overall system. However, it can be envisaged that in cases of the asymmetrical, e.g., one/two hormone:receptor complexes, with different upper/lower arm conformations, the movement of each FnIII-3 and FnIII-3’ domain, and associated TM helices, along the membrane can occur at different rates/speeds for each FnIII-3-TM and FnIII-3’-TM’ group. This, in turn may have varying effects on the ‘unzipping’ of the JMs from the TKs, leading – importantly - to differential, and spatial-temporally diverse kinase activation. For example, this could result in a transient, ‘touch-and-go’ *trans*-phosphorylation involving only a single TK, rather than a prolonged and symmetrical activation of both TKs.

It can also be assumed that a non-symmetrical TK activation, and, ultimately, the degree of the TK’s *trans*-phosphorylation, can be modulated and dictated by the receptor-binding properties of the ligand, i.e., its *k*_on_/*k*_off_ rates. Such on/off rates dependence of functioning of the IRLs have already been proposed (77, 78), and the DFA model tries to provide here a most general mechanistic explanations of these phenomena.

Although the lack of AF3 success in generating FnIII-3-TM-JM-TK dimers was not surprising here, its failing to provide dimers of TKs alone is more puzzling. Does this mean that the TK dimers contacts/*trans*-phosphorylations are very transient (as mentioned above), or do they involve – still structurally unseen – more significant conformational changes in the TK upon receptor activation and their dimerization? Nevertheless, the simplicity of use, fast – minutes – runs of the AF programmes, and, most importantly, their very dynamic development allow to hope for some answers here in the foreseeable future.

Finally, the importance of the heterogenic ‘dragging potential’ proposed here in the DFA model (i.e., speedy for the ECD stems – slow for the intracellular JM-TK) can be amplified further by the very likely intracellular interaction of C-terminal tails of the receptors (and CD domains in the dmIR) with the membrane-underpinning cytoskeleton, different lipids composition, density and fluidity of the membrane and membrane micro-structures (e.g., with or without caveolin) (79, 80), IR-clustering membrane dynamic condensates (81), changes in activities of membrane-lipids sorting flippases (82, 83), and many other physiological processes affecting chemistry and structure of cell membranes.

## Summary

The DFA model outlined here is a very simple attempt to unify structural and biochemical evidences reported for ILRs ectodomains and their intracellular components. Its general character is striving to maintain a holistic approach to the knowledge about the components of the receptors, combining all their elements needed for an effective signal transduction. It cannot/does not pretend to be a final view of signal transduction through the ILRs, but it aims to provoke alternative thinking about this process, highlighting some of its aspects that – so far - eluded unambiguous experimental approaches.

Firstly, the DFA model underlines the importance of the already well postulated JMs domains inhibitory effect on the TKs (35–37), especially extensively investigated and evident in EGFR and other classes of the TK receptors (e.g., the EGFR’s JM is even partially helical being in very close interactions with the cell membrane) (47, 48), (84–86) (87). However, the DAF model expands the JMs’ auto-inhibitory role onto their possible stabilisation of the activated forms of the TKs’ active loops, thus giving the JMs’ dual and opposite functionalities in the activation process of the receptors.

Secondly, the DFA model highlights the likelihood of a very intimate character of the JM/TK interaction with cell membrane: possibly an important feature of receptors’ activation process that relies on the JM/TK:membrane-inner-leaf ‘friction’, hence taming the intracellular movement of the TKs in response to hormone binding in order to allow unzipping of the JMs from their in-TK-groove auto-inhibitory conformation. Different qualitative roles of the protein:protein and protein:lipid interactions for the EGFR-like receptors have already been highlighted (88).

Surprisingly, the DFA model translate molecularly very sophisticated, specific (atomic – ‘quantum-like”) receptors’ ECD:hormone mechano-sensing interactions into activation of their TKs, and the initiation of a chemical signalling, by a very inter-molecularly “crude” (“Newtonian-like”) drag-and-friction forces. However, the distinctive properties of the hormone/analogue, and its interactions with the ILRs-ECDs sites 1/2, remain invariably crucial for the nature of the functionality of the hormone, and its downstream signalling signatures as they dictate the dynamics of TM/JM/TK movements. Interestingly, the emphasis of a possible importance of ‘simple’ mechanical forces as a part of ILRs activation parallels a growing recognition of such interactions, mechanical stress concentrations, and resulting feedback loops, being crucial for such diverse life’s phenomena like formation of embryo and organogenesis, to cancer (89, 90) and geometry of rose’s petals (91).

Finally, the DFA model emphasise the emerging role of the heterogeneity of cell membrane inner/outer leaves, their different chemical composition and structural micro-/macro-diversity in modulation of activation of Insulin-Like Receptors. It also underlines the necessity of more frontal – although methodologically most challenging – attack on the phenomena that occur on outer/inner membrane leaf:receptor interfaces, without understanding of which the insight into the signal transduction through ILRs may remain incomplete.

## Data Availability

The raw data supporting the conclusions of this article will be made available by the authors, without undue reservation.
